# SjTPdb: integrated transcriptome and proteome database and analysis platform for *Schistosoma japonicum*

**DOI:** 10.1186/1471-2164-9-304

**Published:** 2008-06-26

**Authors:** Feng Liu, Ping Chen, Shu-Jian Cui, Zhi-Qin Wang, Ze-Guang Han

**Affiliations:** 1 Shanghai-MOST Key Laboratory of Health and Disease Genomics, Chinese National Human Genome Center at Shanghai, Shanghai, PR China; 2 Shanghai Jiao Tong University School of Medicine, Shanghai, PR China

## Abstract

**Background:**

*Schistosoma japonicum *is one of the three major blood fluke species, the etiological agents of schistosomiasis which remains a serious public health problem with an estimated 200 million people infected in 76 countries. In recent years, enormous amounts of both transcriptomic and proteomic data of schistosomes have become available, providing information on gene expression profiles for developmental stages and tissues of *S. japonicum*. Here, we establish a public searchable database, termed SjTPdb, with integrated transcriptomic and proteomic data of *S. japonicum*, to enable more efficient access and utility of these data and to facilitate the study of schistosome biology, physiology and evolution.

**Description:**

All the available ESTs, EST clusters, and the proteomic dataset of *S. japonicum *are deposited in SjTPdb. The core of the database is the 8,420 *S. japonicum *proteins translated from the EST clusters, which are well annotated for sequence similarity, structural features, functional ontology, genomic variations and expression patterns across developmental stages and tissues including the tegument and eggshell of this flatworm. The data can be queried by simple text search, BLAST search, search based on developmental stage of the life cycle, and an integrated search for more specific information. A PHP-based web interface allows users to browse and query SjTPdb, and moreover to switch to external databases by the following embedded links.

**Conclusion:**

SjTPdb is the first schistosome database with detailed annotations for schistosome proteins. It is also the first integrated database of both transcriptome and proteome of *S. japonicum*, providing a comprehensive data resource and research platform to facilitate functional genomics of schistosome. SjTPdb is available from URL: .

## Background

Schistosomiasis remains one of the most prevalent and serious parasitic diseases, and there are ~200 million patients in 76 countries and territories predominantly in tropical and subtropical regions. Schistosomiasis is caused by the multi-cellular parasite schistosomes, which include three major species – *Schistosoma japonicum*, *S. mansoni*, and *S. haematobium *[1]. S. *japonicum *is endemic in China, the Philippines and some other sites in East Asia. Schistosomes have a complex life cycle, including free-living life stages (cercaria and miracidium) and life stages parasitizing in the snail hosts (sporocyst) or definitive mammalian hosts (egg, schistosomulum and adult). The eggs, produced by adult females that are deposited in the liver, intestines, and other host organs, are the major contributors to the pathology and morbidity associated with schistosomiasis. However, schistosome biology, host-parasite interactions and molecular mechanisms of immunopathology of schistosomiasis are not well understood. Comprehensive genomic, transcriptomic, and proteomic analyses will shed light on these aspects and facilitate the development of novel intervention strategies for the control and treatment of schistosomiasis.

In transcriptomic analysis, expressed sequence tags (ESTs) are useful resources for cataloguing expressed genes. For schistosomes, more than 43,000 and 163,000 ESTs from various life stages of *S. japonicum *[1] and *S. mansoni *[2,3] have been acquired and analysed by our group and others, representing the first gene-discovery program and an initial step towards sequencing the complete genome sequence of this parasite. We identified and characterized 611 *S. japonicum *EST clusters with complete open reading frames (ORFs) in our earlier study [1]. Although ESTs are useful resources for monitoring gene expression, they represent the stretches or fragments of transcripts and usually cover only part of full-length genes. Furthermore, complementary DNAs (cDNAs) are limited in providing expression features, because they do not indicate the subcellular localization and post-translational modifications of proteins. On the other hand, proteomic strategies represent a feasible method to monitor protein profiles and complement transcriptomic strategies. Recently, our group reported an integrated comprehensive transcriptomic and proteomic survey of *S. japonicum *[4]. In that study, we identified ~15,000 EST clusters and 8,420 protein-coding genes including ~3,000 transcripts with entire ORFs. The transcriptomic data of *S. japonicum *were collected from schistosomulum, adult worm (including mix-sex adult worm, male worm and female worm), egg and miracidium (minor EST data) [4]. Moreover, we verified the expression states of ~3,200 genes by proteomics throughout different life stages of *S. japonicum *(all developmental stages except for the sporocyst), tegument samples from mix-sex adult worm, male worm, female worm and schistosomulum, and eggshell. The findings represent a comprehensive transcriptomic/proteomic view of *S. japonicum *and should lead to a more profound understanding of schistosome biology and the host-parasite relationship [4].

However, it should be pointed out that other transcriptomic approaches, such as oligonucleotide microarray, were also used in *S. japonicum *and *S. mansoni *studies [5-11]. Several aspects of schistosome biology have been investigated by these approaches, including differential expressions of genes across different life cycles in *S. mansoni *[5,6] and *S. japonicum *[7], gender-specific expression in *S. mansoni *[8,9] and *S. japonicum *[10], as well as species-specific transcription [11]. These studies provide comprehensive information on growth, development, sex differentiation and maturation of this pathogen. Furthermore, in recent studies, the transcriptome of adult worm of *S. mansoni *has been investigated by serial analysis of gene expression (SAGE) technology [12,13]. Indeed, the report by McKerrow and co-workers perhaps represents the most comprehensive survey to date on the transcriptome of the developmental stages stages of *S. mansoni *including cercariae, juvenile liver-stage worms, adult worms, miracidia, mother sporocysts and eggs [13].

Recently, the *S. japonicum *genome has been sequenced by the Chinese National Human Genome Center at Shanghai, and the sequence datasets, including assembled supercontigs and predicted genes, are publicly available [14]. The sequencing of *S. mansoni *genome was also performed by a genome sequencing consortium of The Institute for Genomic Research (TIGR) and the Wellcome Trust Sanger Institute [15], with sequence data available from their website [16]. The latest assembled version 3.1 of the *S. mansoni *genome has approximately 19,000 supercontigs and ~13,000 predicted full-length genes [17,18]. These genomic data of *S. japonicum *and *S. mansoni *certainly represents a new invaluable resource. However, we feel that for best use in the future of these data the massive information from *S. japonicum *needs to properly organized and curated to enable its most efficient access and investigation. The aim of the present study was to establish a comprehensive database dedicated to *S. japonicum*, encompassing data derived from sequence analysis as well as links to external online resources. The database integrates transcriptomic and proteomic resources of *S. japonicum*, as well as shortcuts linking to online genomic and transcriptomic datasets of *S. japonicum *and *S. mansoni*. We consider that SjTPdb will represent a valuable tool and provide more effective access to a wealth on information on schistosome development, evolution and host-parasite interplay.

## Construction and content

### Data collection

SjTPdb contains (1) 84,499 ESTs of several, discrete developmental stages of *S. japonicum*; (2) 14,962 EST clusters; (3) 8,420 protein-coding genes [4], where these *S. japonicum *genes were annotated in detail in SjTPdb; (4) the proteomic dataset of *S. japonicum*. The draft genomic data of *S. japonicum*, including contig sequences and supercontig sequences, the EST clusters [3] and predicted genes derived from draft genomic data (version 4.0) of *S. mansoni *[16], are publicly available through their website gateways [14].

### EST analysis

All ESTs from *S. japonicum *were first filtered to remove low quality sequences. The remaining ESTs were masked at the both ends for contaminating regions, including vector sequences, adaptor, poly (A/T) and restriction sites. The masked regions of ESTs were changed, not trimmed, into the equal length of poly "N", as this processing would ensure that the length of EST sequences remains the same as their corresponding quality files, so that users can check conveniently the quality of specific regions of selected ESTs. The processes of EST assembly, protein coding sequence (CDS) prediction and the primary annotation have been reported previously [4]. Finally, the available 14,962 EST clusters contain 10,434 contigs and 4,528 singletons, where 8,420 were considered as CDS-containing.

### Annotation methods

The functional annotations of these CDS-containing EST clusters were mainly based on sequence similarity to known genes in public databases [4]. The 8,420 translated peptides were compared by BLASTP with sequences from UniProtKB/Swiss-Prot Release 55.0 [19] and the non-redundant (NR) database from NCBI [20] (downloaded on March-12-2008), respectively. In general, for a given query from these *S. japonicum *proteins or peptides, its BLASTP hit with the lowest E value in the both databases must be accepted for functional annotation. The public databases contain *S. japonicum *protein sequences submitted previously by us. Therefore, to avoid the self-annotations, we discarded all self BLASTP hits and chose the next best BLASTP hit for annotation. However, if the BLAST hit is to a sequence of uncertain function – indicated by descriptions like "hypothetical", "unknown" and "uncharacterized", a meaningful hit was selected. We also performed BLASTX searches using EST clusters against SwissProt database in addition to BLASTP searches. We found a few EST clusters had better hits in reverse strand, suggesting an error in ORF prediction. In these cases, we reversed the sequence and chose the correct ORF according to BLASTX results. We also found some ORFs with apparent frame-shift or truncation mutations involving single neucleotide insertion or deletion. Moreover, we found some EST clusters with hidden intron sequences that usually introduce stop codons. In these cases, the EST clusters were re-assembled and checked manually to eliminate errors. After obtaining the correct protein sequences, we performed BLASTP searches using these new sequences against NR and SwissProt databases and annotated these proteins as above.

The *S. japonicum *protein will be annotated as "hypothetical protein" if its BLASTP E value is higher than E-5. All of the *S. japonicum *genes were annotated as "SJCHGCxxxxx protein, xxx aa, BLASTP (against SwissProt/NR) similarity to xxx, E = xxx, Identities = xxx" or "SJCHGCxxxxx, xxx aa, hypothetical protein".

The functional classification of *S. japonicum *proteins was performed using terms from the Molecular Function and Biological Process aspects of the Gene ontology (GO) system [21]. To examine homologous protein domains, the peptide sequences of *S. japonicum *were used to search the InterPro database [22] by software InterProScan [23], the Pfam database [24] by HMMER 2.4i [25], and the PROSITE database [26] by ScanProsite [27], respectively. Potential signal peptides of *S. japonicum *proteins with full length CDS were identified by SignalP 3.0 [28,29]. Transmembrane helices were predicted with the TMHMM 2.0 server [30] and HMMTOP 2.0 [31]. Putative subcellular localizations of *S. japonicum *proteins were identified by TargetP 1.1 [32] and PSORT II [33].

In the present report, expression levels of *S. japonicum *proteins across different developmental stages, sexes, and within tegument and eggshell were calculated based on our EST and proteomic data, as previously reported [1,3]. In the future, we plan to re-calculate expression levels based on *S. japonicum *gene microarray analyses. To address this issue, we will (1) integrate microarray data reported by others [7,10,11], (2) perform a comprehensive microarray study covering most life stages of *S. japonicum*, and (3) accept submitted microarray data. The expression level calculations will be updated at least once per year to reflect the advances in the study of *S. japonicum *molecular genetics. Genetic variations, including single nucleotide polymorphism (SNP), deletion and insertion (INDEL) of *S. japonicum *genes, and associated and microsatellites have been described previously [4], and in like fashion we will aim to include new information on these phenomena as it becomes available.

### Database implementation

SjTPdb was built on the Windows XP operating system, and the data are parsed into a MySQL database that is accessible publicly via the Apache web server. The PHP and Perl programming languages are used to produce dynamic web pages in respons to users' queries. A local BLAST program is incorporated into SjTPdb, including BLASTP, BLASTN, BLASTX, TBLASTN and TBLASTX. A pull-down menu for database selection is included, with a "NR/NT" option where users can perform BLAST searches against the current non-redundant (NR) protein database or the nucleotide sequence database of NR (NT) of GenBank. When the option is selected, the web page will be transferred automatically to the BLAST interface in NCBI. In addition, we have embedded links in SjTPdb for BLAST searching against *S. mansoni *EST data and genomic sequences of *S. japonicum *and *S. mansoni*.

## Utility and discussion

### Database overview

*S. japonicum *is a multi-cellular parasite of mammals including humans and also represents a model member of the phylum Platyhelminthes. Previous comprehensive 'omics surveys have produced valuable information for schistosome biology [4]. In SjTPdb we have integrated different types of expression data of *S. japonicum *including transcriptomic and proteomic profiles across the developmental stages of this flatworm. Although the ESTs and some *S. japonicum *gene sequences are accessible in public databases, there is only minimal biological annotation available. Herein, SjTPdb provides the biological annotation for these nucleotide sequences in much more detail, which will facilitate further data mining. This can be anticipated to lead to better understanding of schistosome biology.

An important feature of SjTPdb is the incorporation of proteomic data. The proteomic data contains the expression profiles of cercaria, miracidium, tegument and eggshell that complements transcriptomic data [1,3]. It is the first genomic database of schistosome that integrates both transcriptomic and proteomic datasets.

**Figure 1:**
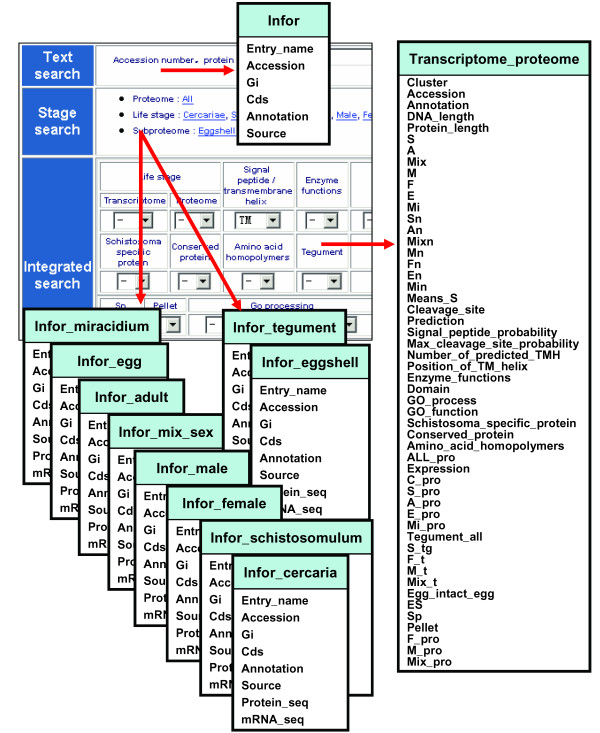
**Schema of SjTPdb, an integrated transcriptome and proteome database of *S. japonicum***. The field names of each MySQL databases in SjTPdb are shown. The database "infor" and "transcriptome and proteome" can be queried through text search and integrated search, respectively. The other databases can be queried by stage search.

The schema of SjTPdb is shown in Figure 1.

### Annotated pages for *S. japonicum *genes/proteins

In SjTPdb, each gene sequence entry of *S. japonicum *encoding proteins is well-annotated. A typical annotation page contains the following information:

*Entry information*: basic entry information such as protein name, accession number(s) (protein) and gene identifier (gi) with a clickable link to GenBank.

**Figure 2:**
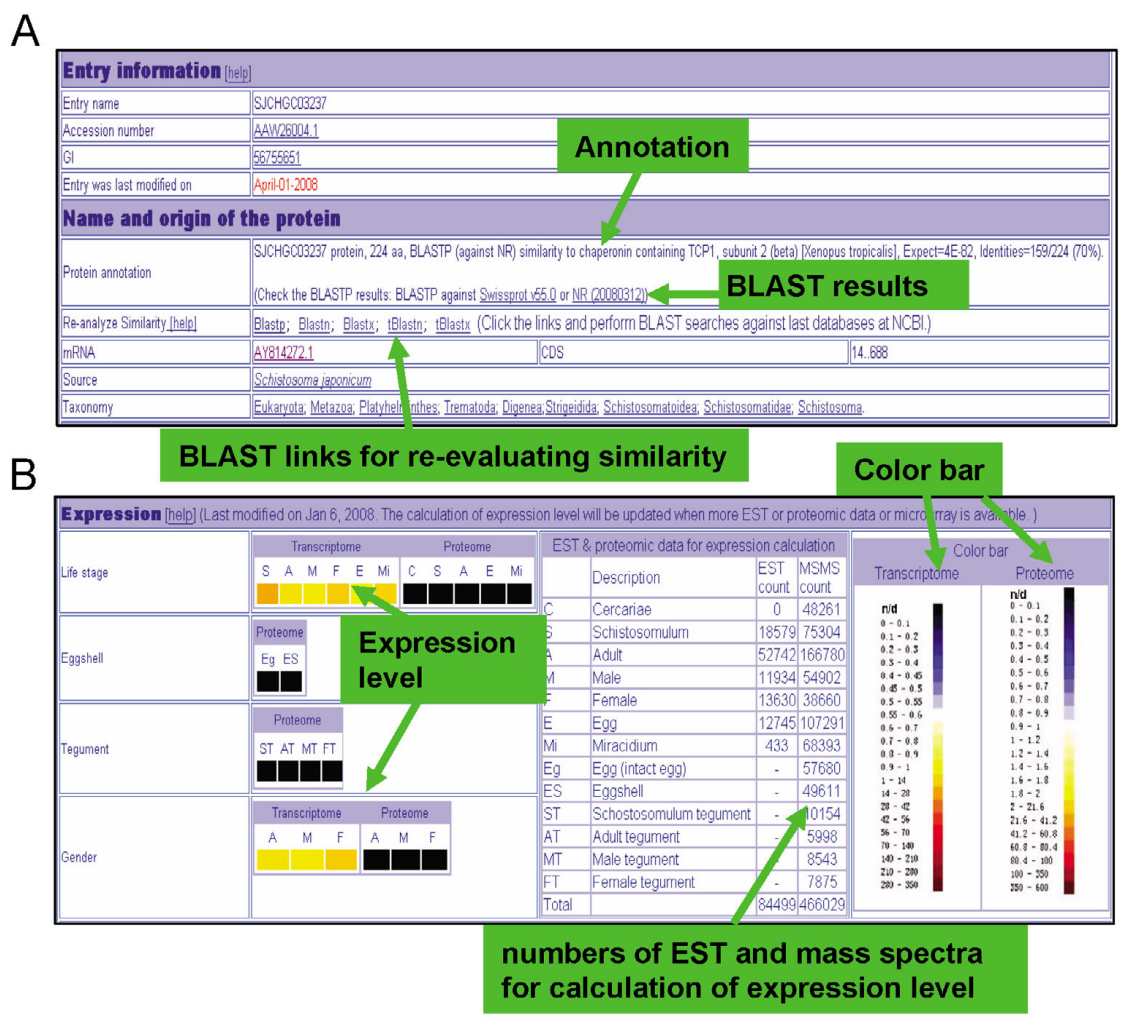
**The annotation page of a *S. japonicum *protein**. A, The head of annotation page for a sample protein in SjTPdb, showing five BLAST links for re-analysis of similarity comparison of this protein against external public databases. B, The expression patterns of a *S. japonicum *protein across different life cycle stages, gender and tegument or eggshell. The expression levels are represented by changing colors according to the legend of the color bar (right).

*Name and origin of the protein*: this section lists a brief biological annotation of this protein based on the similarity to known proteins, as well as the mRNA accession number, CDS region and taxonomy. For a given annotation, the details of BLAST similarity are shown in this section as an indication of the confidence evaluation of the annotated gene. Users can check the detailed BLAST result files via the links. The similarity comparison, however, could become outdated when the public database is updated, leading to false annotations. To re-evaluate the similarities, we offer users five clickable BLAST links, which under clicking will submit the protein or DNA sequence automatically to BLAST engines in NCBI (Figure 2A) and users can perform BLAST searches against the latest public databases.

References: citations of available publications reporting the generation and analysis of the *S. japonicum *gene/protein are provided.

*Gene Ontology*: functional categories by Gene Ontology associations are listed here if assigned. Again, users can re-analyze the GO assignments by clicking the quick link embedded in the page.

*Family and domain*: this section records conserved protein motifs or domains of the *S. japonicum *protein, obtained by comparing with Interpro, Pfam and Prosite databases.

*Molecular characteristics*: this section provides information on predictions on whether the *S. japonicum *protein is secreted, anchored to the membrane, or located in other subcellular organelles, based on predictions by SignalP, TMHMM, HMMTOP, TargetP and PSORT programs using artificial neural networks, hidden Markov methods (HMM), k-nearest neighbours classifier [34] or other methods. Secreted proteins released by *S. japonicum *may function as enzymes in invasion [35] or feeding process [36], or as immune modulators [37], while the membrane proteins may contribute to signal transduction, immune evasion, antigen mimic, and host-parasite interaction [38]. For brevity, only the summarized prediction result is displayed; detailed descriptions can be obtained through clickable internal links.

*Genetic polymorphisms*: genetic diversity of schistosome populations, determined as SNPs, INDELs and microsatellite allelism may lead to the variations in infectivity, drug sensitivity, pathogenicity, immunogenicity, host species range and so on. Genetic diversity is apparent among 1,496 of 8,420 *S. japonicum *genes, involving at least 6,038 known SNPs, with 2,272 synonymous and 1,552 nonsynonymous substitutions found in the CDS regions [4]. Furthermore, a small subset nonsynonymous SNPs were verified by mass spectrometry analysis [4]. The genetic polymorphisms could significantly confound the host immune responses targeting these antigens, which will need to be considered during development of new vaccines and drug targets.

*Expression*: schistosomes are dioecious and the sexually mature adults display major dimorphism, with distinct male and female forms. Investigations on gender-enriched expression can be expected to elucidate mechanisms of development, sexual maturation and egg production in schistosomes [39,40]. To obtain the expression differences between sexes, we calculated the expression levels of mixed-sex, male and female adult worms based on both transcriptomic and proteomic analyses (Figure 2B). Trancript expression levels were calculated using relevant EST numbers, while the protein abundance were represented by the ratio of the sum of all Mascot peptide scores for the protein to the total number of tandem mass spectra of the protein [4,41]. The results were transformed into colors for intuitive visualization, with the legend displayed in the protein pages.

The schistosomes have complex developmental cycles that have evolved to include adaptation to free-swimming aquatic environment, intermediate host snail in an aquatic environment and warm-blooded mammalian host environment. Characterization of expression patterns across these life stages of *S. japonicum *will provide further insight into schistosome biology, molecular mechanisms of immunopathology of schistosomiasis and host-parasite interplay [6,42]. Here, we further calculated and displayed the expression patterns of the *S. japonicum *cercaria, schistosomulum, egg and miracidium with the integrated information from both transcriptomic and proteomic datasets (Figure 2B). While the transcriptome expression profile of the cercaria was not included because the ESTs of this life stage were not collected, the expression pattern of cercariae is represented here at least by proteomic data.

The outer covering of the schistosome, the tegument, is a living syncytial layer; it is evident on the schistosomulum and the adult, stages that inhabit the human blood vessels. The tegument plays a pivotal role in nutrient uptake [43-45] and locates at the interface of the parasite and its host. Thus tegumental proteins represent potential drug targets and vaccine candidates. Here, we presented the expression patterns of predicted tegumental proteins on each annotation page based on our earlier studies of the protein expression profiles in schistosomula, mixed-sex adults, and male and female adults of *S. japonicum *[4].

The egg of schistosome is covered by a sclero-proteinaceous eggshell within which the miracidium develops [46]. Eggshell proteins may contribute to initiation of host immune response [47]. For each eggshell protein, we present its eggshell expression pattern on the annotation page, with its expression in intact eggs shown for comparison.

*Sequence information*: this section shows both the protein and the encoding DNA sequence of a given gene/protein, which are downloadable as compressed files. Shown on the top of the sequences are the length, deduced molecular weight (MW) and isoelectric point (pI) of the protein.

### Querying the database

**Figure 3:**
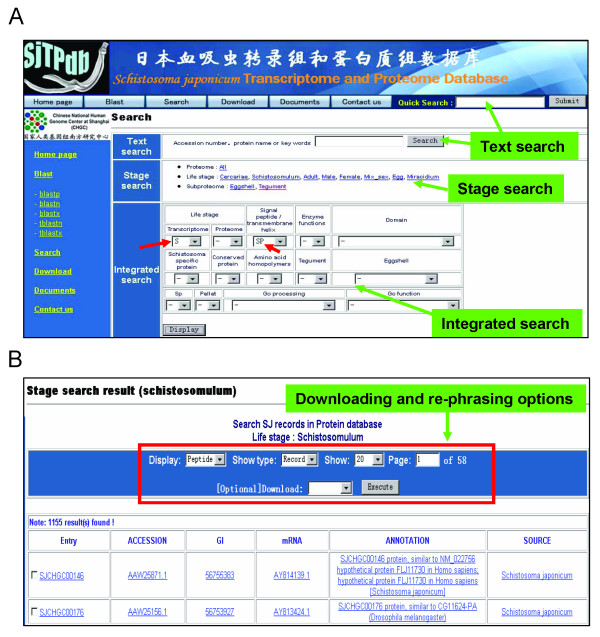
**Search interface and the search results**. A, The search interface of SjTPdb is illustrated, showing search tools by the text, life cycle developmental stage and integrated selections. The red arrows indicate the selections of a combined search for the putative secreted proteins with the detectable transcripts in the schistosomulum stage of the parasite. B, an example of a query result, with options for downloading and re-phrasing.

SjTPdb offers users four types of web-based tools to query and download information from the database – text-based, life stage, integrated, and BLAST search programs. The text-based tool is used to retrieve the annotation pages for a given gene/protein by keywords such as protein name, accession number, EST cluster name, or any words that exist in the annotation line (Figure 3A). The query result can be displayed in a table form with each hit listed in a row. However, because the output page is configurable, users can set the display options to show the mRNA or protein sequences in fasta format. Also, users can specify the number of rows for display when the querying result contains more than 20 rows (default number). The output table includes six columns that provide links to the relevant gene or protein annotation pages in SjTPdb, in addition to outer links to related proteins and mRNAs deposited in GenBank. Furthermore, the query result and the related protein or DNA sequences are downloadable as plain tables and fasta files (Figure 3B), respectively. Users can also choose to download part of the result by checking the boxes in the front of each row.

The life stage search provides users with a shortcut to retrieve the specialized expression profiles of a given developmental life stage of *S. japonicum*, as represented in the proteomics datasets [4]. This search also offers direct access to profiling differential gender-associated proteins and sub-proteomes of the parasite's eggshell and tegument.

Unlike text-based or life stage searches, the integrated search provides a convenient and flexible way to retrieve specific information on particular topics of *S. japonicum *by employing multiple constrains. For example, when "TM" option is selected within "Signal peptide/transmembrane helix" section, the output will display the records of *S. japonicum *proteins with putative transmembrane domains. When both "TM" and "ES" options within "Eggshell" section are checked, the query result will exhibit the putative transmembrane proteins detected in eggshell samples. Similarly, users can retrieve a specific subset of information of *S. japonicum *by applying more constrains. This function is expected to be especially useful for in-depth data-mining and to obtain more fundamental insights into schistosome biology. Furthermore, the output format of the query result by this search tool has more detailed information than that of the text-based and life stage searches. Here, the output tables and the related DNA or protein sequences are downloadable freely.

**Figure 4:**
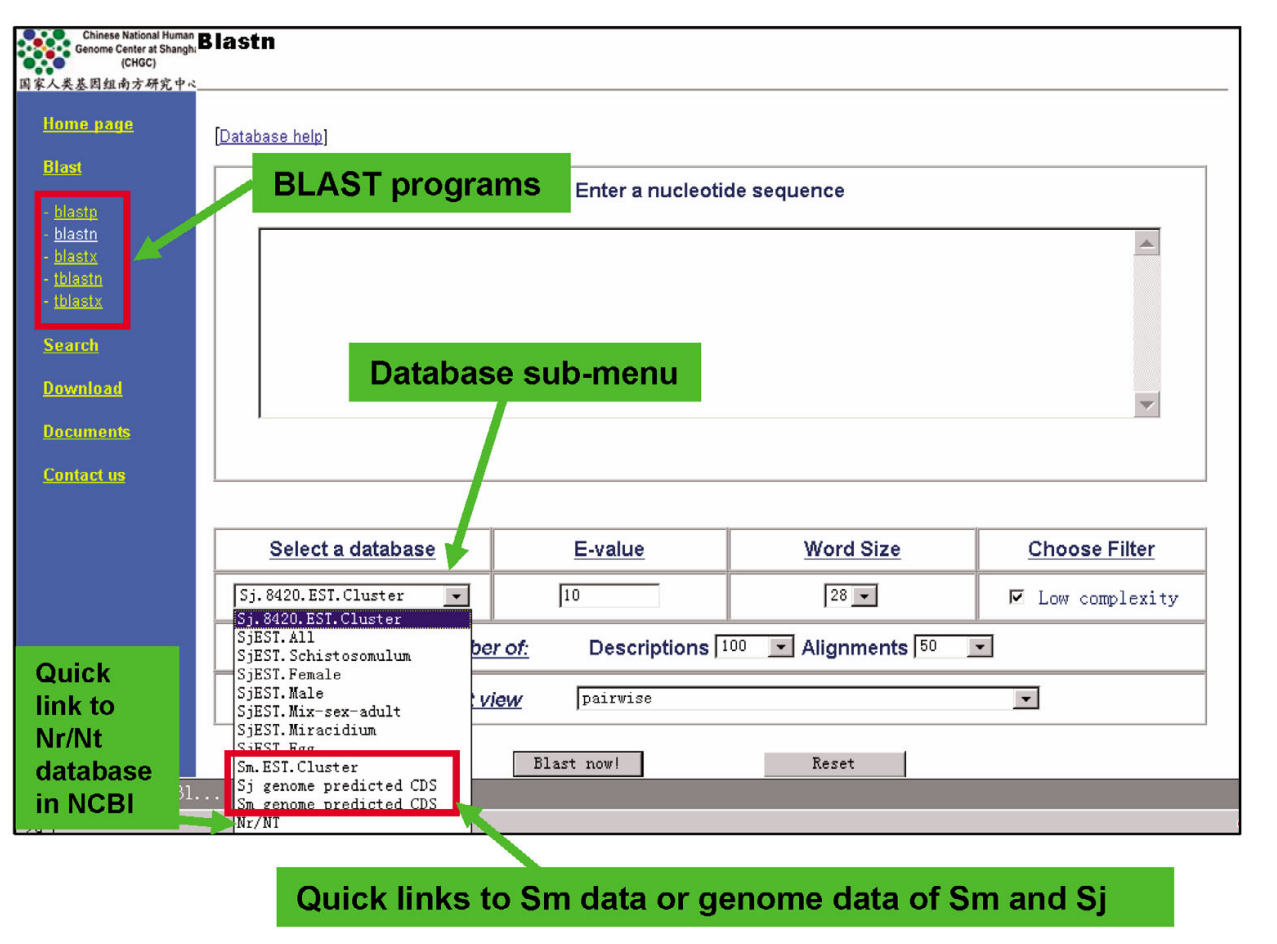
**BLAST search tools implemented in SjTPdb**. We implemented five kinds of BALST programs in this database. To perform similarity comparison, users can choose various sequence databases of schistosome and external links to NR/NT databases at NCBI within the database sub-menu.

BLAST search tools are powerful and used widely for sequence similarity comparison. We implemented BLAST algorithms in SjTPdb (Figure 4). Herein, the searchable sequences in SjTPdb include EST/EST cluster sequences, protein sequences deduced from EST clusters and the predicted genes of *S. japonicum *and *S. mansoni*. If users fail to locate significant hits in schistosome databases, they can search against the more comprehensive NR or NT databases (Figure 4).

All *S. japonicum *sequences in SjTPdb are freely downloadable, along with the EST sequences of *S. japonicum *from the discrete developmental stages and the corresponding quality files of EST reads. The information also includes names of ESTs incorporated into each 14,962 EST cluster and tables with the association links between EST accession numbers and EST clones.

### Future prospects

The databases will be regularly updated to reflect future progress in genome, transcriptome and proteome analyses of *S. japonicum *and other schistosomes. The expression patterns of genes across developmental stages of *S. japonicum *will be reconstructed using microarray data of published studies or ongoing projects. We are also planning to launch a comprehensive SNP scanning analysis of *S. japonicum *genome. New data will be incorporated into the SjTPdb, providing an even more comprehensive data-mining platform for investigation on schistosome biology and, hopefully, to facilitate development of new interventions for schistosomiasis.

## Conclusion

In SjTPdb, we have integrated comprehensive transcriptomic and proteomic datasets of a human blood fluke, *S. japonicum*, and implemented convenient tools for querying and data retrieving. We consider that SjTPdb represents an important contribution to the genomics of *S. japonicum*, and we expect it will facilitate fundamental research on schistosome evolution, development, host-parasite interplay and so forth.

## Availability and requirements

The database is located at  and is suitable for most graphical web browsers. All sequences can be freely downloaded from SjTPdb (see Download section).

## Authors' contributions

FL conceived the study, designed and optimized the website, and drafted the manuscript. PC constructed the database and performed the computational analysis. S–JC and Z–QW contributed data to the database. Z–GH supervised the work and revised the manuscript.
